# Synthesis and Characterization of Activated Carbon-Supported PdRu Nanoparticles Decorated with Different Proportions of Co for Ammonia-Borane Hydrolysis

**DOI:** 10.1021/acsomega.5c02174

**Published:** 2025-07-23

**Authors:** Rawezh Muhtasim Mustafa, Hilal Çelik Kazıcı

**Affiliations:** † Faculty of Engineering, Department of Chemical Engineering, 125665 Koya University, 45001 Koysinjaq, Iraq; ‡ Faculty of Science, Department of Chemistry, 53000 Van Yüzüncü Yıl University, 65080 Van, Turkey

## Abstract

In this study, palladium (Pd), ruthenium (Ru), and cobalt (Co) nanoparticles (NP) supported on activated carbon (AC) (denoted as PdRu/AC@1% Co) were successfully synthesized via the impregnation–reduction method. The structural and surface characteristics of the catalyst were thoroughly analyzed by using X-ray diffraction (XRD), scanning electron microscopy (SEM), energy-dispersive X-ray spectroscopy (EDX), and X-ray photoelectron spectroscopy (XPS). The catalytic performance of PdRu/AC@1% Co with different atomic ratios was evaluated for hydrogen generation through the hydrolysis of ammonia borane (NH_3_BH_3_, AB). Compared to monometallic (Pd) and bimetallic (PdRu) systems, the trimetallic PdRu@Co catalyst exhibited significantly enhanced catalytic activity, even at low temperatures. Under optimized conditions (1 mmol of AB, 50 mg of catalyst, 25 °C), the highest turnover frequency (TOF) value of 312.8 (mol_H_2__)/(mol_cat_·min) was achieved. The activation parameters for the hydrolysis reaction were calculated as activation energy (*E*_a_) = 19.6 kJ/mol, enthalpy change (Δ*H* ^#^) = 17.12 kJ/mol, and entropy change (Δ*S* ^#^) = −184.85 J/(mol·K). These findings suggest that PdRu/AC@1% Co is a highly efficient and reusable catalyst, making it a promising candidate for practical hydrogen production from AB.

## Graphical abstract

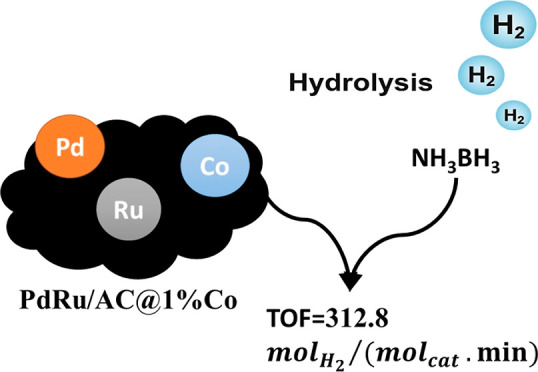

## 1 Introduction

Due to increasing concerns about environmental pollution and overreliance on fossil fuels, hydrogen energy has gained significant attention from researchers and scientists in recent years. Global issues such as water pollution, the energy crisis, and greenhouse gas emissions have raised alarms and necessitate urgent solutions.^1^ As a result, there has been growing interest in renewable energy sources and efficient energy storage technologies.^2–6^ Hydrogen (H_2_) is an ideal sustainable clean energy source for the future due to advantages such as its lightness, abundance, and high energy density.^7^ It offers significant potential to meet the increasing global energy demand.^8–10^ The reliability and efficiency of the materials and technologies used to store hydrogen are other important features of hydrogen energy systems.^10^ Among various hydrogen storage materials, ammonia borane (NH_3_BH_3_, AB) has attracted great interest due to its high hydrogen content (19.6 wt %), low molecular weight (30.86 g mol^–1^), and good stability.^11–13^ The complete hydrolysis of 1 mol of AB can theoretically produce 3 mol of hydrogen gas, making it an efficient candidate for hydrogen generation (eq 1).^14–18^

1 $$H_{3}{NBH}_{3}+2H_{2}O\to {NH}_{4}^{+}+{{BO}_{2}}^{-}+3H_{2}$$

Compared to other borohydrides such as sodium borohydride (NaBH_4_), which also serves as a hydrogen storage material with a hydrogen content of about 10.8 wt %, AB offers several advantages. NaBH_4_ typically requires alkaline conditions and elevated temperatures to release hydrogen effectively, which can limit its practical applications.^19^ In contrast, AB releases hydrogen efficiently under milder, often near-neutral conditions, facilitating more convenient and safer catalytic hydrolysis processes.^20^ Additionally, AB demonstrates better thermal stability and lower toxicity compared to NaBH_4_, which further supports its potential for on-demand hydrogen generation in fuel cells and portable energy devices. However, challenges related to controlling hydrogen release rates and managing reaction byproducts remain active research areas.^21^ These factors make AB a particularly attractive hydrogen storage candidate within the broader field of hydrogen storage materials.

Heterogeneous catalysis using metal nanoparticles is a well-established method for the controlled release of hydrogen from AB.^22^ Developing nanoparticle-based catalysts with high surface activity and enhanced stability is therefore crucial for efficient hydrogen production.^23^ Nanoparticles exhibit unique catalytic properties due to their high surface-to-volume ratio, quantum confinement effects, and synergistic interactions between multiple metal species.^24,26^ Various noble metal-based catalysts such as Pd,^17,27–29^ Pt,^30,31^ Ru,^32–37^ and Rh^38,39^ have been studied for AB hydrolysis. However, their limited availability and high cost pose significant challenges to their large-scale applications.^40^ Accordingly, developing noble metals and cost-effective non-noble-metal-based composites is critical. In particular, cobalt-based catalysts are an important choice for catalyst design because of their significant benefits, which include low cost, strong stability, and high catalytic activity.^41–43^ Moreover, we hypothesize that the incorporation of Co into the PdRu/AC catalyst system enhances the electron transfer between Pd and Ru atoms, thereby promoting a synergistic catalytic effect. The presence of a third transition metal such as Co can modify the electronic environment of active metal sites, improving adsorption–desorption behavior and facilitating faster hydrogen generation kinetics. Such trimetallic combinations have been reported to optimize the adsorption energy of intermediates and significantly improve catalytic performance compared to their monometallic or bimetallic counterparts.^44^ Accordingly, the Co-decorated Pd_0_._5_Ru_0_._5_/AC catalyst is expected to demonstrate superior hydrogen evolution activity in the hydrolysis of AB. Furthermore, catalyst support materials and the size of metal nanoparticles are critical factors influencing both the catalytic performance and durability in repeated use. It is widely acknowledged that multimetallic nanoparticles often outperform monometallic ones due to synergistic interactions among different metal components.^45–49^ Due to their high surface energy, metallic nanoparticles tend to aggregate, especially in multimetallic systems where interactions between different metal species can promote particle agglomeration, which adversely affects catalytic activity. The use of stabilizing support materials helps to prevent such aggregation during the preparation of metallic nanostructures.^50–53^ Carbon-based materials, especially AC, are commonly used as supports due to their high surface area, porosity, electrical and thermal conductivity, chemical stability, adsorption capacity, and cost-effectiveness.^54–57^ These properties make AC an excellent support for developing stable and active nanocatalysts.^25^

This study focuses on the design and performance evaluation of trimetallic PdRu/AC@Co catalysts for the catalytic hydrolysis of AB under various experimental conditions. The surfaces of the prepared catalysts were characterized by techniques such as SEM–EDX and XPS. It was aimed to determine the optimum effects of AB (NH_3_BH_3_) and its concentration, temperature, catalyst amount, and metal loading ratio on the hydrolysis reaction, which are the most important parameters affecting the hydrogen production of the PdRu/AC@%1 Co catalyst. In addition, kinetic data related to the catalytic hydrolysis of AB under optimum conditions, parameters such as Δ*H* ^#^, Δ*S* ^#,^ and Ea, were obtained.

## 2 Experimental Section

### 2.1 Reagents

Potassium tetrachloropalladate (II) (K_2_PdCl_4_, 99.99%), ruthenium­(III) chloride trihydrate (RuCl_3_·3H_2_O, 99.9%), cobalt­(II) chloride hexahydrate (CoCl_2_·6H_2_O, 98%), ammonia borane (NH_3_BH_3_, 97%), sulfuric acid (H_2_SO_4_, 97%), and sodium borohydride (NaBH_4_, 98%) were purchased from Sigma-Aldrich. Purified water obtained by using the HUMAN ZeneerPower I system was used in all experiments.

### 2.2 Preparation of Pd/AC and PdRu/AC Catalysts

AC-supported catalysts were prepared using the impregnation–reduction method. This approach was intentionally selected to achieve better control over the metal dispersion and intermetallic interactions. Sequential impregnation helps prevent agglomeration and unwanted alloying that could negatively impact catalytic activity, thus promoting a more effective synergistic effect among Pd, Ru, and Co.^19^ Pd/AC, PdRu/AC, and PdRu/AC@Co catalysts K_2_PdCl_4_, RuCl_3_·3H_2_O, and CoCl_2_·6H_2_O were prepared by using metal precursor salts. The Pd/AC catalyst, 0.1 g of the AC support material, and 0.0307 g of K_2_PdCl_4_ were sonicated in distilled water, and then the resulting slurry was stirred at 500 rpm for 3 h. An aqueous solution of NaBH_4_ was then slowly transferred into the suspension to reduce the Pd^2+^ ions dispersed on the AC surface. The resulting solid precipitate was centrifuged after being rinsed three times with ethanol and water. The washed solid was dried in a vacuum oven at 60 °C for 12 h.

Similarly, the PdRu/AC catalyst, 0.0307 g of K_2_PdCl_4_, 0.0192 g of RuCl_3_·3H_2_O metal salts, and 0.1 g of AC were dispersed together in deionized water and stirred for 3 h. NaBH_4_ was added to reduce the number of ions on the AC surface. After washing in distilled water and ethanol, it was dried at 60 °C for 12 h.

### 2.3 Preparation of PdRu/AC@*X* Co (*X*: 1%, 3%, 5%, 7%, 10%) Catalysts

To examine the effect of cobalt NP addition on the catalytic hydrolysis reaction, the PdRu/AC catalyst was decorated with different percentages of cobalt NPs. PdRu/AC@*x*Co nanocatalysts were synthesized by chemical reduction with an optimized PdRu fixed atomic ratio (Pd_0.5_Ru_0.5_) and cobalt contents of 1%, 3%, 5%, 7%, and 10%. 0.1 g of PdRu/AC (0.1 g) and CoCl_2_·6H_2_O (0.022 g) were sonicated in deionized water and mixed at 500 rpm for 3 h. A NaBH_4_ solution was added in a controlled manner. Thus, Co nanoparticles were stabilized on the PdRu/AC surface. After washing in distilled water and ethanol, it was dried at 60 °C for 12 h.

### 2.4 Characterization of the Catalyst

XRD trends of the crystal structures of the compounds were reported with a Rigaku D/max-220/PC X-ray diffractometer D/max-220/PC with CuK5–007 radiation (40 kV, 20 mA). SEM and EDX analyses were performed to investigate the morphology and structure of the substance collected. SEM images were captured using ZEISS SIGMA 300 microscopes functioning at acceleration rates of 20 and 10 kV. The electron binding structures of Pd, Ru, and Co on the catalytic surface were studied using the Specs-Flex electron spectrometer and XPS.

### 2.5 Hydrogen Production Test

The catalytic hydrolysis of AB was carried out in a three-necked glass vessel connected to a gas buret to measure the amount of H_2_ produced by the hydrolysis reaction, as previously described.^58^ A 50 mg portion of the catalyst was dispersed well in 5 mL of ultrapure water and poured into the reaction vessel. An aqueous solution of AB (100 mM, 30.8 mg) was then added to the reactor (rotating at 600 rpm). The reaction was carried out in a circulating water bath at 298 K. The volume of hydrogen produced from the start of the reaction was recorded at equal time intervals, and the catalyst efficiency was graphically shown. The effects of AB amount (mM), catalyst amount (mg), temperature, and %Co loading rate on catalytic hydrolysis experiments were studied under different conditions. To evaluate the catalytic performance of the catalyst system in the hydrolysis of AB, kinetic studies were conducted at various temperatures under identical experimental conditions. The rate constants were determined based on the initial hydrogen generation rates measured at each temperature. These constants were then analyzed using the Arrhenius and Eyring models to extract activation parameters, such as activation energy, enthalpy, and entropy. Linear regression of the respective plots was used to derive these parameters, providing insight into the reaction mechanism and the influence of the temperature on catalytic efficiency. For the reusability test, after the completion of the first reaction cycle, one equivalent of the initial amount of AB was added to the reaction medium, and the amount of H_2_ released was recorded. This process was repeated 6 times.

## 3 Results and Discussion

### 3.1 Characterization of the Catalysts

XRD analysis was performed to identify the crystalline phases present in the catalysts. The experimental diffraction patterns were compared with standard reference patterns from the JCPDS database to assign the observed peaks, Figure 1. As for AC (Figure 1a), the XRD pattern demonstrated two broad and major peaks positioned at 2θ = 24.8° and 2θ = 43.4°, related to (002) and (100) planes, respectively,^58^ indicating an amorphous structure of AC. Moreover, a slight shift in the diffraction peaks of AC was observed after the deposition of metal nanoparticles (Figure 1b).^59^ Although the observed shifts are relatively small and not clearly distinguishable in the XRD profiles, such variations may indicate weak electronic or strain-induced interactions between AC and the deposited metal nanoparticles. Similar phenomena have been reported in other studies where carbon-based supports induce minor lattice distortions upon metal deposition.^19,60^

**Figure 1:**
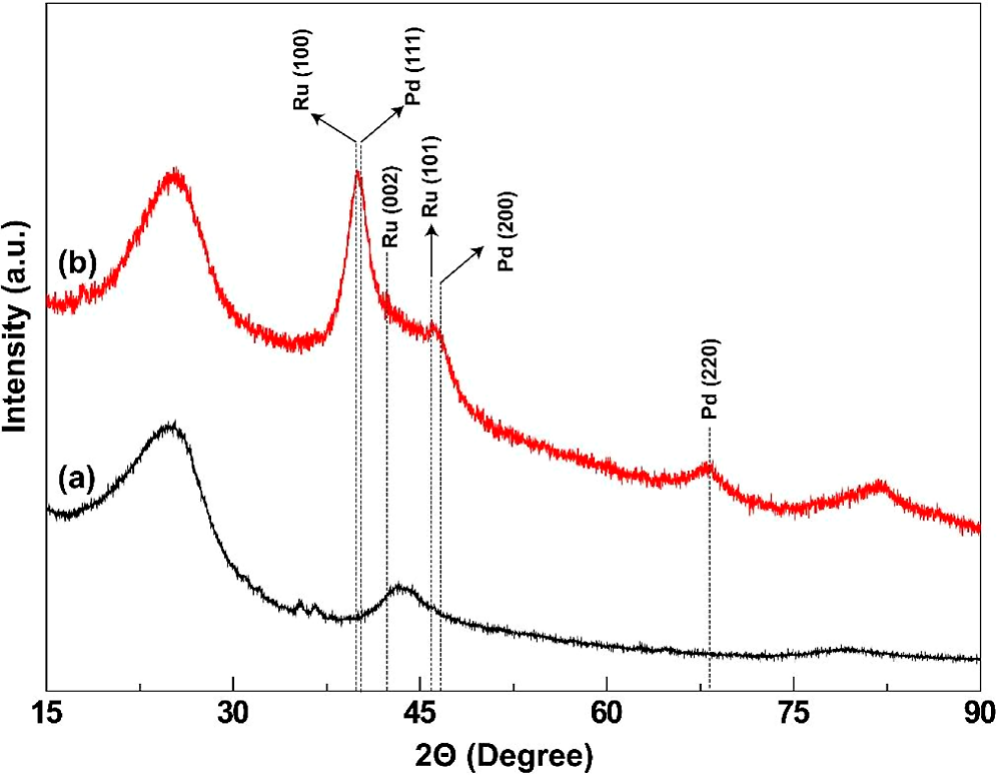
XRD patterns of AC (a) and PdRu/AC@1% Co (b).

Furthermore, the characteristic diffraction peaks observed at 2θ = 40.2°, 46.6°, and 68.2° were assigned to the (111), (200), and (220) planes of face-centered-cubic Pd^0^ (JCPDS 46–1043), while peaks at 2θ = 38.6°, 42.2°, and 45.5° were attributed to the hexagonal phase of Ru^0^ (JCPDS 00-006-0663). However, considering the close proximity of Pd(200) and Ru(002) peaks near 42°, and the potential for alloying between Pd and Ru, it is difficult to definitively distinguish between individual Pd^0^ and Ru^0^ phases solely based on XRD. The possibility of Pd–Ru alloy formation cannot be ruled out and may contribute to the observed peak positions. Similar XRD patterns indicating Pd–Ru alloy formation have been reported previously.^61^ Meanwhile, no characteristic diffraction lines of Co^0^ could be detected, most probably due to the low deposition amount of Co^0^ nanoparticles.^62^ Note that some diffraction lines of Pd and Ru nanoparticles were slightly shifted to higher angles as compared to pure Pd^0^ or Ru^0^, supremely indicating a Pd to Ru and Co substitution.^63^

The surface morphology of the catalysts was examined by SEM, and the corresponding images are shown in Figure 2. The metal nanoparticles appear to be reasonably well dispersed on the AC support, although some degree of agglomeration is observed, especially in the bimetallic and trimetallic samples. This agglomeration is likely due to the high surface area-to-volume ratio of the nanoparticles and possible metal–metal interactions during synthesis. Notably, the incorporation of Co in the PdRuCo/AC catalyst led to more pronounced clustering and surface roughness compared to those in the Pd/C and PdRu/AC samples, suggesting a significant morphological change. Such structural differences can be attributed to strong synergistic interactions between Co and the noble metals (Pd and Ru), which have been reported to modify the electronic structure and promote more active catalytic interfaces. Similar observations were recently reported by Meng et al., who demonstrated that cobalt incorporation into noble-metal catalysts not only alters surface morphology but also enhances catalytic activity in the hydrolysis of AB. While some agglomeration is observed, it should be noted that this phenomenon is influenced not only by the high surface area-to-volume ratio or particle size but also strongly depends on the support characteristics and synthesis methods.^62^ For instance, several studies report that well-dispersed small nanoparticles can be obtained by using functionalized supports or optimized impregnation techniques, which minimize agglomeration and enhance catalytic performance. Moreover, agglomeration generally reduces the number of accessible active sites, negatively impacting catalyst activity.^64^ In our case, although agglomeration is present, the catalytic activity remains high, possibly due to the synergistic effects and strong interactions among Co, Pd, Ru, and the activated carbon support.

**Figure 2:**
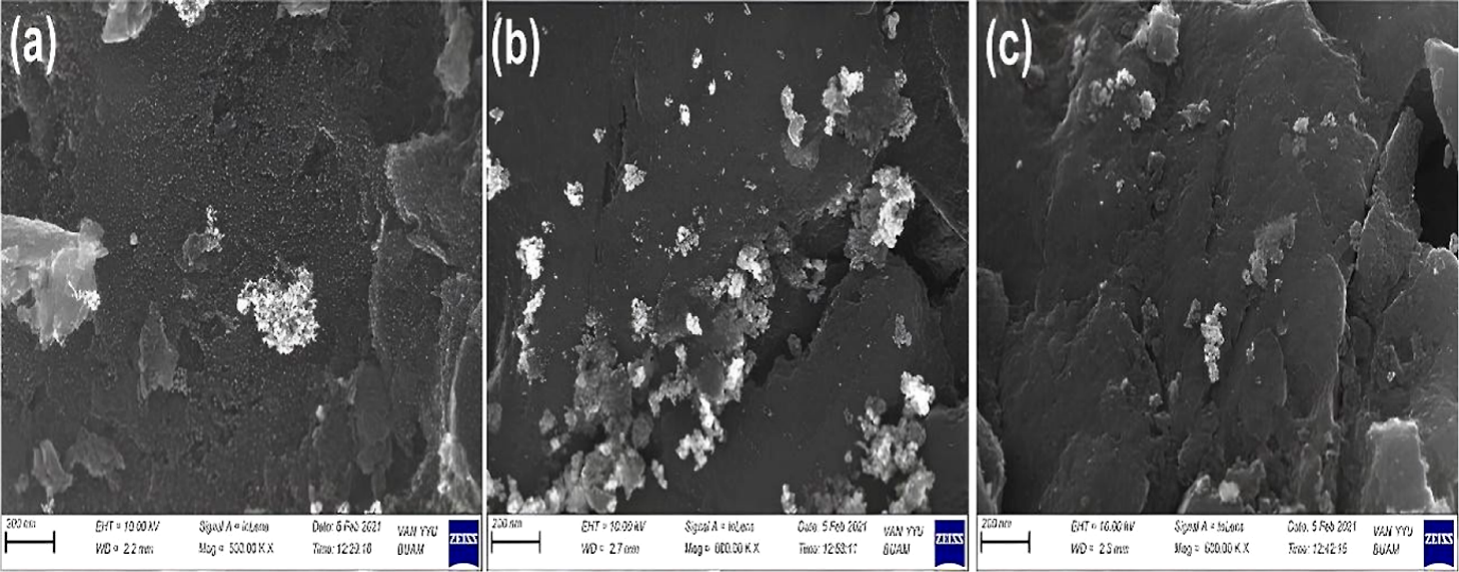
SEM photomicrographs of (a) Pd/AC, (b) PdRu/AC, and (c) PdRuCo/AC.

SEM–EDX and elemental mapping analyses were also carried out for the best catalyst (PdRuCo/AC) in the study. As shown in Figure 3, AC was fully and homogeneously coated with PdRuCo nanoparticles. Moreover, the specific peaks for Pd, Ru, and Co were also detected, and the peaks well matched with the XPS results.

**Figure 3:**
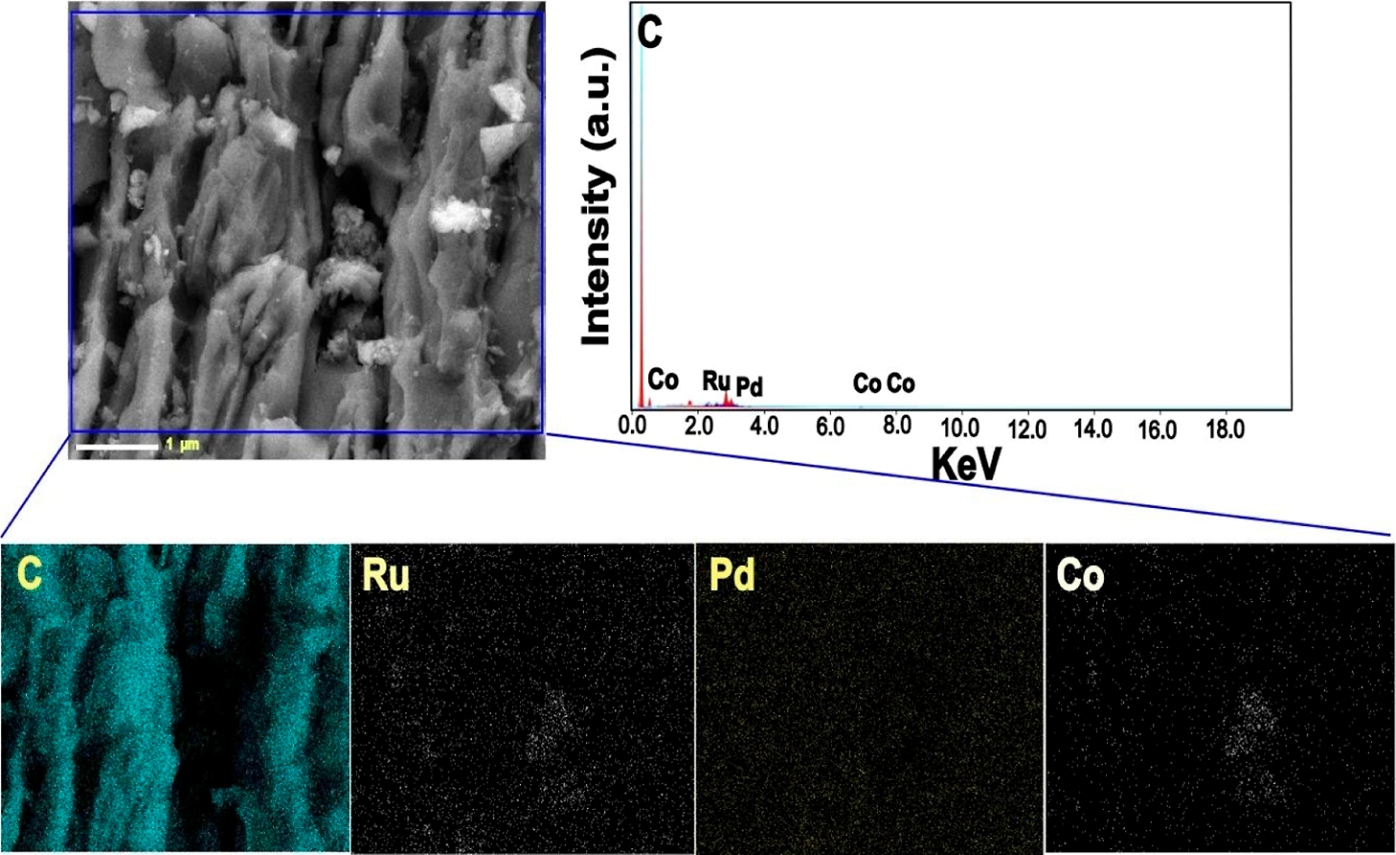
SEM photomicrograph, elemental mapping, and EDX spectrum for PdRuCo/AC.

The surface composition and chemical states of the PdRuCo/AC catalyst were examined via XPS, and the results are presented in Figure 4. The survey spectrum (Figure 4a) confirms the successful deposition of Pd, Ru, and Co on the AC support. High-resolution spectra reveal the presence of metallic Pd^0^ (335.4 and 340.9 eV for Pd 3d_5_/_2_ and Pd 3d_3_/_2_, respectively) (Figure 4b), Ru^0^ (462.4 and 484.3 eV for Ru 3p_3_/_2_ and Ru 3p_1_/_2_, respectively) (Figure 4c), and Co^0^ (780.4 and 796.1 eV for Co 2p_3_/_2_ and Co 2p_1_/_2_, with satellite peaks at 785.4 and 803.7 eV, respectively) (Figure 4d).

**Figure 4:**
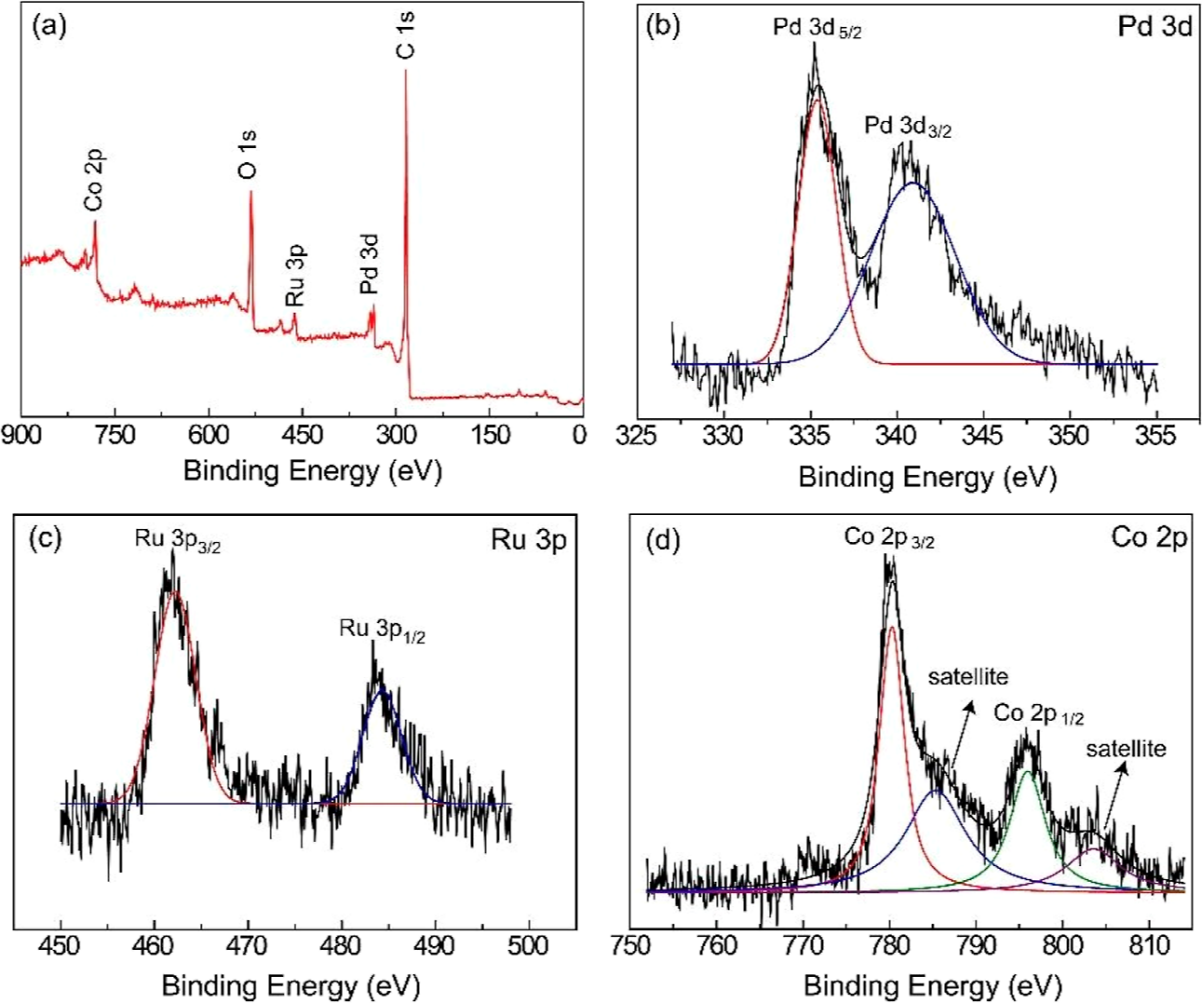
(a) XPS survey scan spectrum of PdRuCo/AC and core-level XPS spectra for Pd 3d (b), Ru 3p (c), and Co 2p (d).

Importantly, the incorporation of Co significantly influences the electronic environment of Pd and Ru. The observed binding energies, particularly the shifts in the Pd 3d and Ru 3p peaks compared to monometallic references, suggest a strong electronic interaction between Co and the noble metals. Such interactions can modulate the electron density around the active sites, potentially enhancing the catalytic efficiency by optimizing the adsorption and activation of reactant molecules. This synergistic effect is consistent with previous findings,^65^ which reported that Co-promotion in Pd- or Ru-based catalysts improved hydrogen generation performance through electronic modulation and enhanced metal dispersion. Thus, Co not only contributes as an active component but also tunes the overall electronic structure of the bimetallic system, which is critical for efficient ammonia borane hydrolysis.

In general, changing the composition, atomic ratio, and mass content of the metal can significantly change the properties of alloy nanoparticles. In this respect, the effect of the developed single, double, and triple metal-based catalysts on the hydrolysis of AB was examined with Pd/AC, PdRu/AC, and PdRu/AC@Co nanoparticles. As a result of the investigation, it was found that complete conversion occurred in the trimetallic catalyst system in the hydrolysis reaction of 100 mM AB and it was more active than the mono and bimetallic catalyst systems (Figure 5). Therefore, it was observed that the trimetallic PdRu/AC@Co catalyst exhibited more effective catalytic activity than the monometallic Pd/AC and bimetallic PdRu/AC catalysts in the hydrolysis reaction of AB. By using the PdRu/AC@Co catalyst, hydrogen was completely released within 40 s and a theoretical hydrogen yield was obtained.

**Figure 5:**
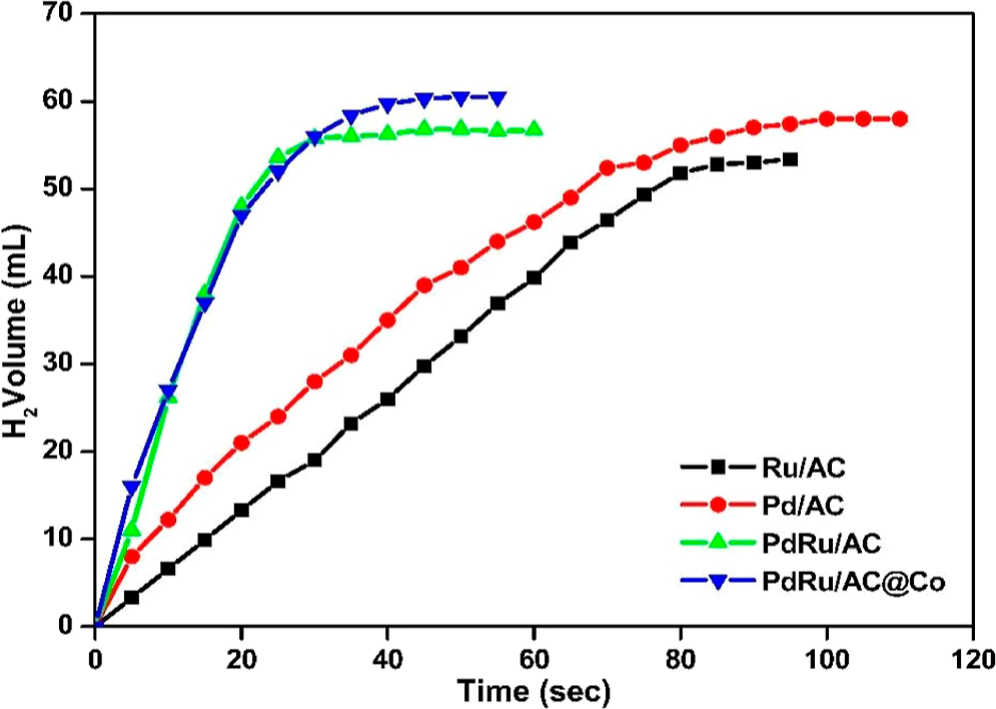
Volume of hydrogen gas (H_2_) released over time during the catalytic hydrolysis of 100 mM AB at 298 K, using monometallic, bimetallic, and trimetallic catalysts supported on AC.

Literature supports the synergistic effect of Co with noble metals in bimetallic and trimetallic systems. Cobalt can modulate the electronic structure of adjacent Pd and Ru atoms, optimizing the adsorption and activation of borohydride species and the release of H_2_. Moreover, Co can improve the metal dispersion on carbon supports, increase the number of accessible active sites, and stabilize smaller particle sizes. These features collectively contribute to faster kinetics. Additionally, previous studies have shown that Co-based trimetallic catalysts, such as PdRuCo or PtCoNi, exhibit improved catalytic performance due to synergistic and ensemble effects between the transition metals.^66,67^ To clarify the specific role of cobalt in the catalytic system, preliminary experiments with activated carbon-supported cobalt nanoparticles (AC@Co) were performed. The results indicated that Co alone exhibits negligible catalytic activity for ammonia borane hydrolysis under the conditions tested, consistent with previous studies.^19^ Consequently, the present work focuses on the synergistic interactions within the PdRuCo trimetallic system.

The effect of the molar ratio of catalysts greatly affects the hydrolysis of the AB. It is aimed to determine the dehydrogenation of the AB in the liquid phase by using catalysts having metal ratios Pd_0.5_Ru_0.5_/AC, Pd_0.9_Ru_0.1_/AC, Pd_0.1_Ru_0.9_/AC, Pd_0.7_Ru_0.3_/AC, and Pd_0.3_Ru_0.7_/AC. The highest hydrogen production rate was obtained using Pd_0.5_Ru_0.5_/AC (Figure 6a).

**Figure 6:**
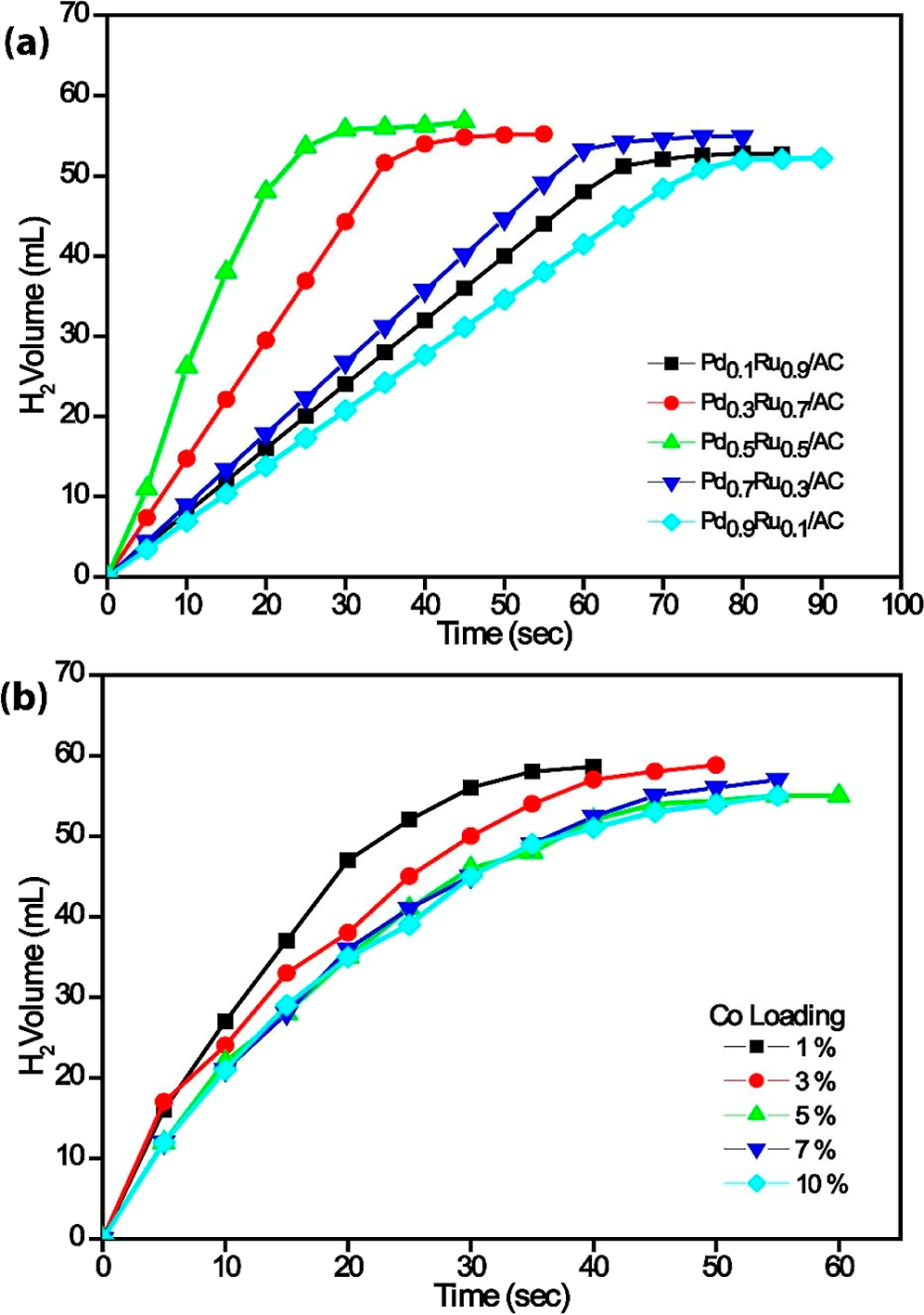
Volume of H_2_ produced over time during the catalytic hydrolysis of 100 mM AB at 298 K. (a) Comparison of the catalytic activity of catalysts with different metal compositions supported on AC and (b) the effect of varying cobalt loadings on the hydrogen evolution rate using Pd_0_._5_Ru_0_._5_/AC@*x*% Co catalysts.

For the effect of cobalt on catalytic activity, catalysts were prepared by using different Co percentages. For this purpose, PdRu/AC@*X* Co (*X*: 1%, 3%, 5%, 7%, 10%) catalysts containing different percentages of Co were investigated for hydrogen production from AB hydrolysis at fixed substrate concentration and room temperature. It was determined that Pd_0.5_Ru_0.5_/AC@1% Co was more effective in terms of total conversion and activity parameters compared to other percentage compositions (Figure 6b). In further experimental studies, the best catalyst, Pd_0.5_Ru_0.5_/AC@1% Co, was used.

The structural and morphological characterizations of the Pd_0.5_Ru_0.5_/AC@1% Co catalyst, which was found to be the catalytically most active material in the hydrolysis of AB, were performed. The Pd_0.5_Ru_0.5_/AC@1% Co catalyst, which was structurally and morphologically characterized, was used in detailed kinetic studies for the hydrolysis reaction of AB.

The effect of the amount of the catalyst on the reaction rate of the AB hydrolysis reaction was investigated by using different amounts of the catalyst (10–100 mg) while keeping the AB concentration constant at 100 mM and the temperature at 298 K. The total gas volume (mL) and time (sec) graph released as a result of the AB hydrolysis reaction are given (Figure 7a). The hydrogen production rate was calculated by determining the slope measured in the nearly linear part of the graph for each Pd_0.5_Ru_0.5_/AC@1% Co catalyst concentration. The line *y* = 1.02*x* + 5.82 is obtained from the graph of hydrogen production rate versus initial concentration of Pd_0.5_Ru_0.5_/AC@1% Co, both in logarithmic scale (Figure 7b). This line graph shows that the catalytic hydrolysis reaction is first order concerning the catalyst concentration.

**Figure 7:**
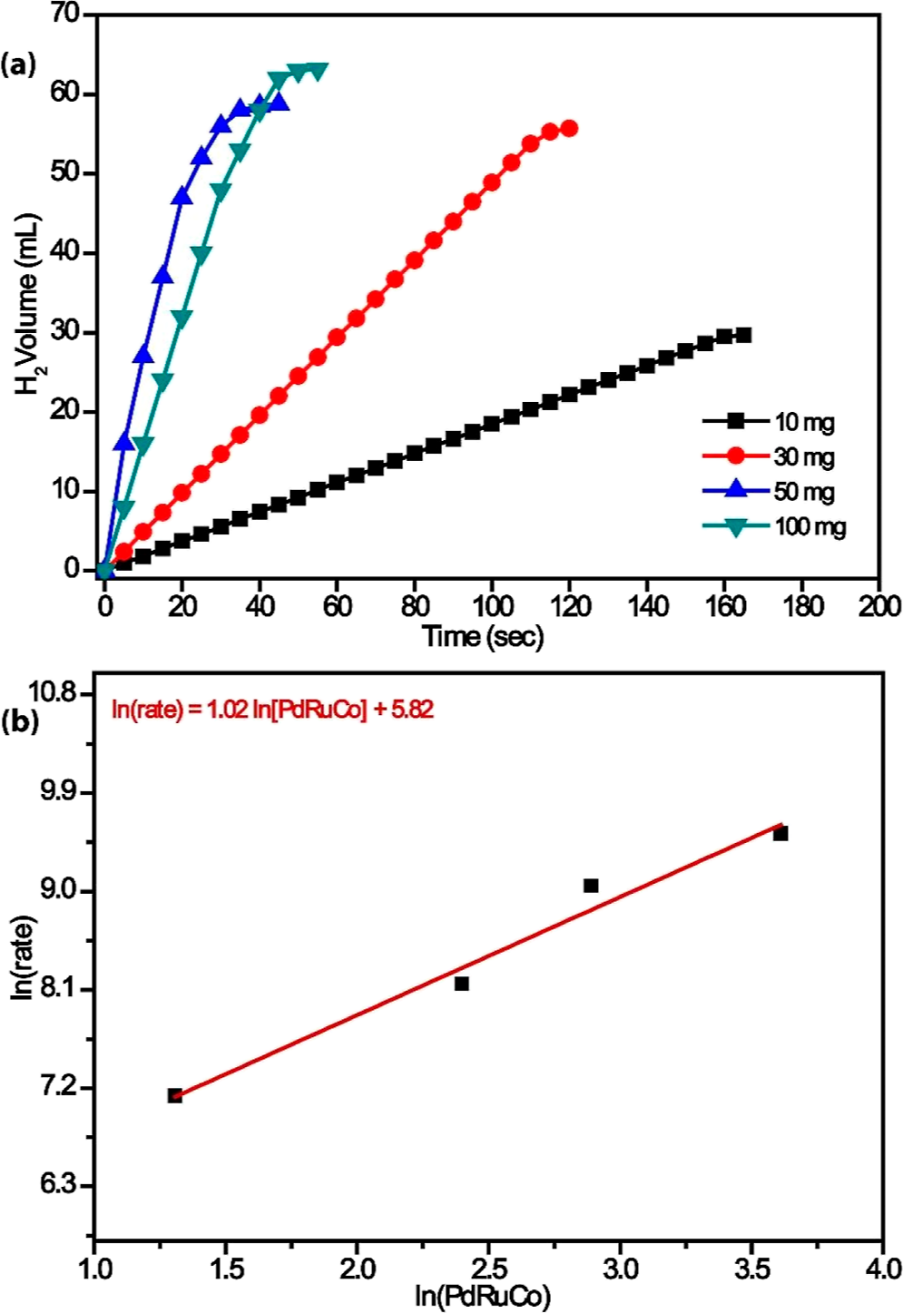
Plots of (a) H_2_ volume evolved over time during the catalytic hydrolysis of 100 mM AB at 298 K with varying amounts of the Pd_0_._5_Ru_0_._5_/AC@1% Co catalyst (10, 30, 50, and 100 mg) and (b) initial hydrogen production rate versus catalyst concentration on a logarithmic scale, illustrating the catalytic performance and kinetics.

The unreactive species are converted to their active forms during the induction period. Then, rapid hydrogen generation starts and continues almost linearly until the consumption of all ammonia borane present in the solution. The hydrogen generation rate was found to be directly proportional to the amount of Pd_0.5_Ru_0.5_/AC@1% Co. For all tests, a complete hydrogen release (mol H_2_/mol H_3_NBH_3_ = 3) was observed.

The effect of the AB substrate concentration on the hydrogenation rate was studied by performing a series of experiments starting with different initial AB concentrations, keeping the catalyst amount constant at 50 mg and the temperature at 298 K. Figure 8 shows the graph of the gas volume released due to AB hydrolysis versus time at different initial AB concentrations (50–300 mM). It can be seen that the slopes of the rate curves do not linearly accommodate ammonia borane concentration in the reaction medium, which has a negligible effect on the rate of hydrogen generation. The rate of hydrogen generation from catalytic hydrolysis of ammonia borane is independent of AB concentration, as evidenced by almost matching slopes of near-linear sigmoidal curves. The hydrogen generation rate was determined from the linear portion of each plot. For all tests, a complete hydrogen release was observed.

**Figure 8:**
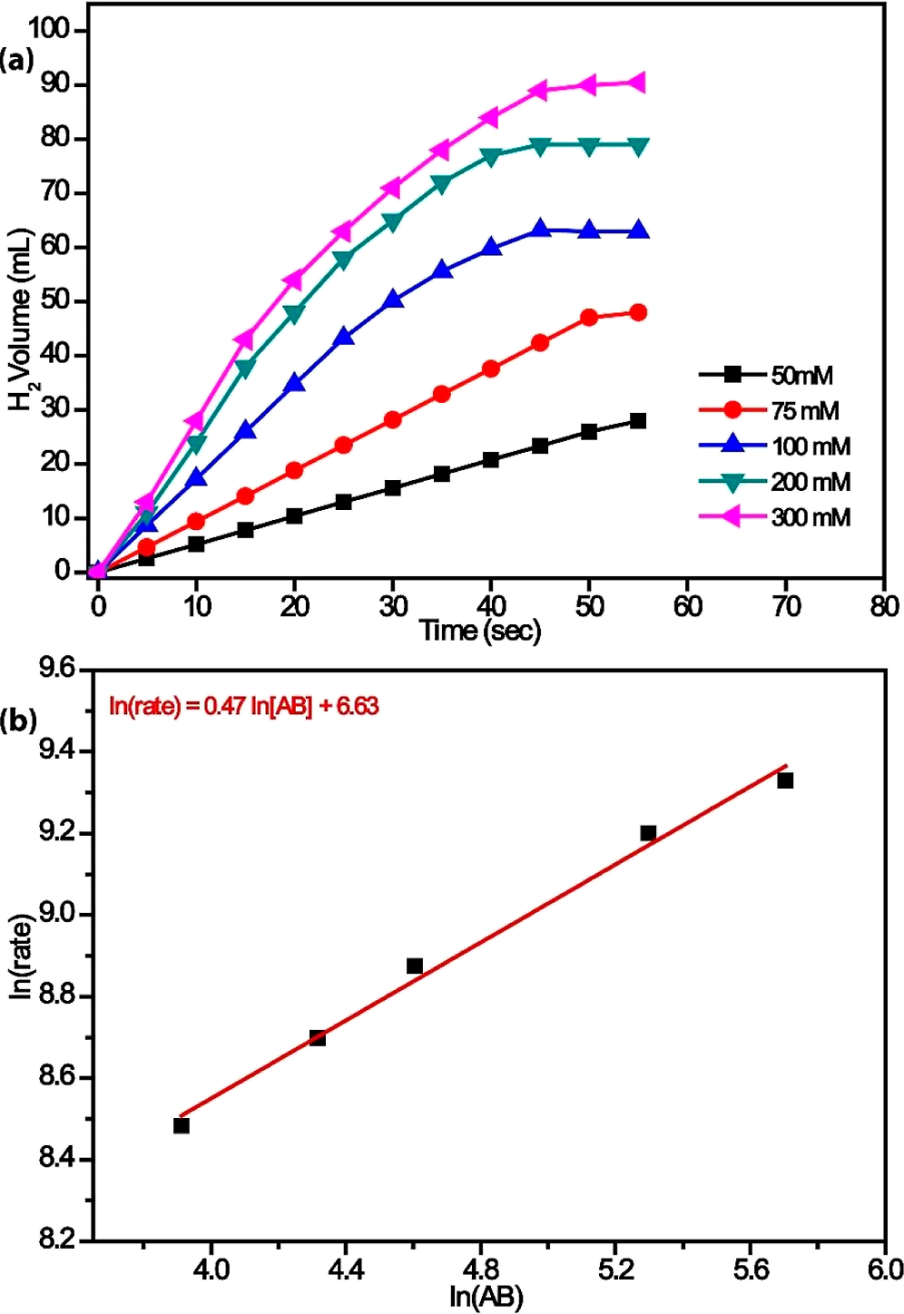
Plots of (a) H_2_ volume evolved over time during the catalytic hydrolysis of AB at 298 K using a fixed catalyst amount of 50 mg and varying initial AB concentrations (50–300 mM), illustrating the effect of substrate concentration on hydrogen generation kinetics and (b) the corresponding plot of initial hydrogen production rate versus AB concentration on a logarithmic scale.

The correct equation *y* = 0.47*x* + 6.63 was obtained from the In­(rate)–In­(AB) graph, taking into account the initial speeds of the reactions. According to the slope obtained from this line graph equation, it can be assumed that the reaction degree of AB is zero.

The effect of temperature on the reaction rate of the AB hydrolysis reaction was investigated using 100 mM AB and 50 mg of the catalyst. In addition, hydrolysis reactions were carried out with the Pd_0.5_Ru_0.5_/AC@1% Co catalyst at different temperatures to calculate the activation parameters (*E*_a_, Δ*H* ^#^, Δ*S* ^#^) of the AB hydrolysis reaction. The total gas volume (mL) and time (s) graph released as a result of these reactions is given (Figure 9). It is observed that the hydrolysis reaction rates of AB increase with the increase in temperature. This increase is a possible expected situation.

**Figure 9:**
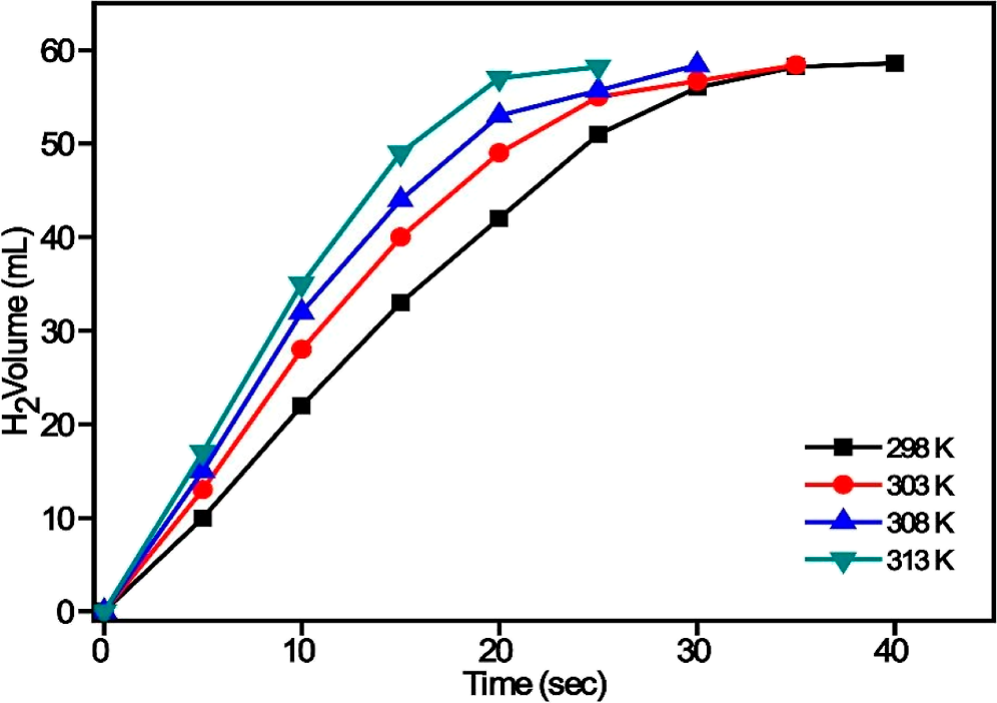
Plots of H_2_ volume evolved over time during the catalytic hydrolysis of 100 mM AB at various temperatures using 50 mg of the Pd_0_._5_Ru_0_._5_/AC@1% Co catalyst, illustrating the effect of temperature on the reaction kinetics.

The rate constant (*k*) values at each temperature were calculated from the rate slopes of the AB hydrolysis reaction catalyzed by the Pd_0.5_Ru_0.5_/AC@1% Co catalyst (Table 1). For each experiment, the total amount of active metal (Pd + Ru + Co) present in the catalyst was precisely determined to be 1.0047 × 10^–5^ mol, as confirmed by ICP-OES analysis. The rate constant was calculated using eq 2

2 $$k=\frac{H_{2}}{mol_{cat}\cdot t}$$

where H_2_ is the amount of hydrogen generated (mol), mol_cat_ is the molar quantity of the active metal species in the catalyst, and *t* is the reaction time (sec.). The initial rates were obtained from the slope of the linear region in the hydrogen volume versus time plots (see Figure 9).

**Table 1:** Effect of Temperature on the Rate Constant of H_2_ Generation from AB Catalyzed by Pd_0.5_Ru_0.5_/AC@1% Co

temperature (K)	rate constant, *k* (H_2_/(mol_cat_·t))
**298**	0.057216
**303**	0.059756
**308**	0.068086
**313**	0.083739

The rate constant/temperature data were evaluated according to the Arrhenius equation and the Eyring equation to obtain the *E*_a_, Δ*H* ^#^, and entropy Δ*S* ^#^ of activation. First, the Arrhenius equation was used for the evaluation (eq 3). With this equation, the activation energy can be calculated from the constant velocity

3 $$\ln k=\ln\left(Ae^{-E_{a}/RT}\right)$$

in the equation, *A* and *E*_a_ are constant properties of the reaction, and *R* is the gas constant. It is assumed that the graph of (In *k*) concerning (1/*T*) is the slope (−*E*_a_/*R*), and the frequency factor (ln *A*) is the intercept of the graph. From the Arrhenius plot and equation, *E*_a_ is equal to 19.65 kJ/mol (Figure 10a).

**Figure 10:**
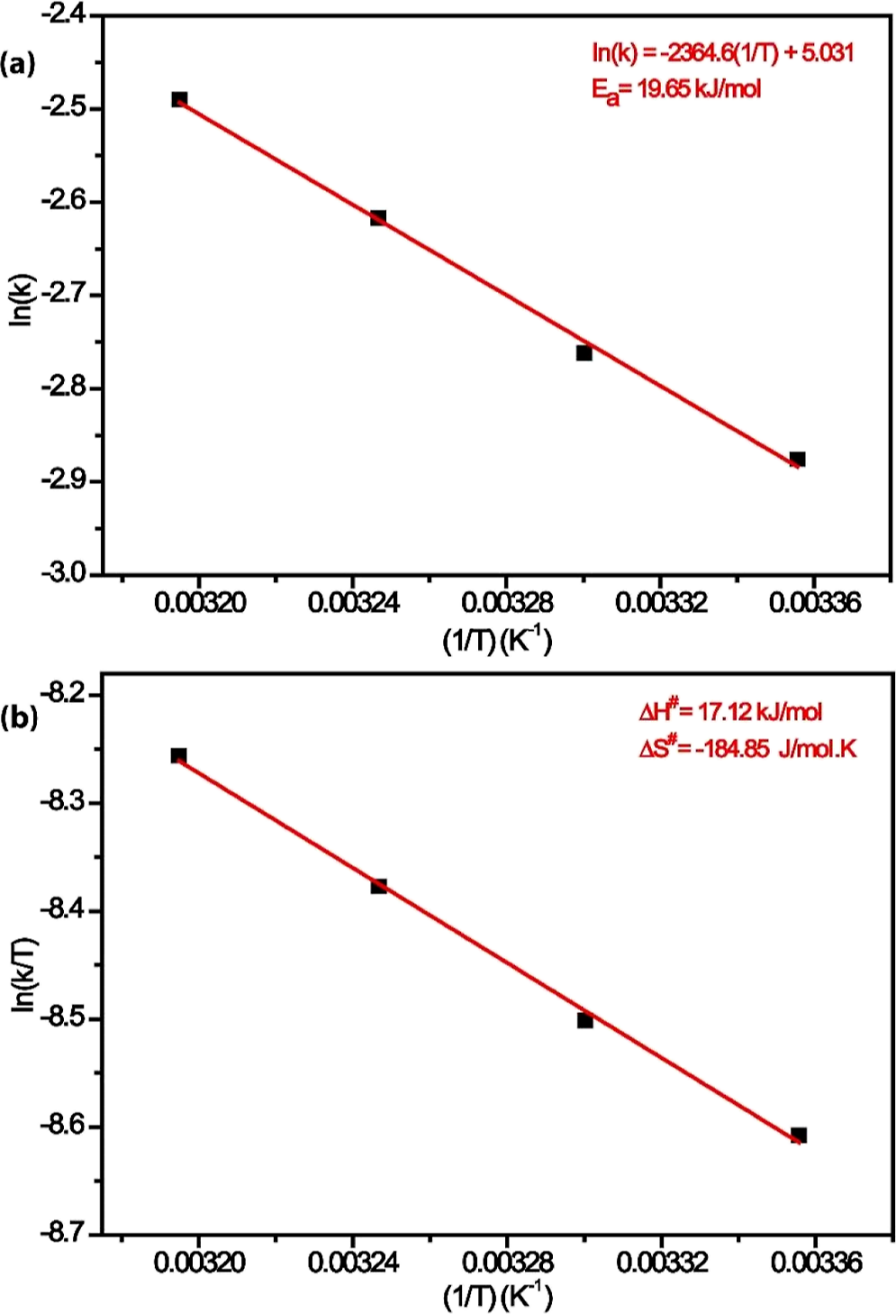
Arrhenius plot (a) depicting the natural logarithm of the hydrogen generation rate versus the reciprocal of temperature (1/*T*) for the catalytic hydrolysis of AB, used to determine the *E*_a_. Eyring–Polanyi plot (b) showing ln­(*k*/*T*) versus 1/*T*, employed to calculate the Δ*H* ^#^ and Δ*S* ^#^ parameters for the reaction catalyzed by Pd_0_._5_Ru_0_._5_/AC@1% Co.

Other important activation parameters, Δ*H* ^#^ and Δ*S* ^#^, were found by the Eyring equation (eq 4).

4 $$\ln\left(\frac{K}{T}\right)=\frac{1}{T}\left(-\frac{\Delta H}{R}\right)+\ln\left(\frac{K_{b}}{h}\right)+\frac{\Delta S}{R}$$

The slope of the graph of ln (*k*/*T*) versus (1/*T*) is (−Δ*H*/*R*), where *R*: gas constant ratio, *K*_b_: Boltzmann constant, *h*: Planck constant, and the frequency factor (ln­(*K*_b_/*h*) + (Δ*S*/*R*)) is the intercept of the graph. Figure 10b shows the Eyring graph (plot of absolute temperature (1/*T*) vs ln (*k*/*T*)). The slope of the straight line gives an enthalpy of activation of 17.12 kJ/mol, and the intercept gives an entropy of activation of −184.8 J/(mol·K). The small value of activation enthalpy and large negative value of activation entropy are indicative of a correlative mechanism for AB hydrolysis catalyzed by the Pd_0.5_Ru_0.5_/AC@1% Co catalyst, which is consistent with the mechanism proposed for the hydrolysis of ammonia borane given in the literature.

Turnover frequency (TOF) is the number of moles per unit of time that each active site on the catalyst surface converts reactants to the target product, consistent with methodologies used in similar multimetallic catalytic systems. TOF is the most fundamental idea for determining the effectiveness of a catalyst by measuring the number of active sites participating in the reaction.^65^ For trimetallic systems, we considered the total metal loading determined via ICP-OES analysis, and TOF was calculated according to the following (eq 5)­

5 $${TOF}_{initial}=\frac{n_{H_{2}}}{n_{cat}\times t}$$

where $n_{H_{2}}$ is the number of moles of hydrogen generated, $n_{cat}$ is the total number of moles of active metals in the catalyst, and *t* is the reaction time in minutes for complete conversion.

TOF for hydrogen production from the hydrolysis of AB was determined from the hydrogen production rate in the linear part of the graph plots in the presence of the Pd_0.5_Ru_0.5_/AC@1% Co catalyst. The TOF value of the Pd_0.5_Ru_0.5_/AC@1% Co catalyst at 298 K in AB hydrolysis was calculated as 312.8 (mol_H_2__)/(mol_cat_·min). *E*_a_ and TOF values obtained in various studies reported in the literature are given in Table 2. In this study, it is seen that both the activation energy (19.65 kJ/mol) and the TOF value of the catalyst reported are better and more efficient than many studies reported in the literature. As a result of comparing the catalytic activity and activation parameters of various catalysts mentioned in the comparison table, it was determined that the developed Pd_0.5_Ru_0.5_/AC@1% Co catalyst is a potential candidate for AB hydrolysis.

**Table 2:** Comparison of Kinetic Parameters and TOF Values of Various Catalysts for AB Hydrolysis, along with Reaction Temperature and Substrate Concentration

catalyst	Δ*H* ^#^ ^a^	Δ*S* ^#^ ^b^	*E*_a_ ^c^	TOF^d^	*T* ^e^	[AB]^f^	ref
NP-Ru			66.5	26.7	298	100	^32^
Ru(0)/TiO_2_			70	241	298	100	^33^
Ru/γ–Al_2_O_3_			23		323	280	^34^
Ru/C	42.3	141.8	76	113	298	260	^66^
RGO/Pd			51	6.25	298	300	^67^
Co_35_Pd_65_/C			27.5	22.7	298	200	^68^
Ag@Co/graphene			20.03	102.4	298	200	^69^
Ag@Ni/graphene			49.56	77	298	200	^69^
p(AMPS)-Co/Ni	30.37	196.96	33.05	49	298	50	^70^
Pt(%8)/CCF-500			39.2	35	303	50	^71^
Ni@meso-SiO_2_			29	18.5	298	200	^72^
***Pd***_***0.5***_ ***Ru***_***0.5***_ ***/AC@1%*** Co	***17.12***	***184.85***	***19.65***	***312.8***	***298***	***100***	***this work***

a Activation enthalpy (kJ/mol).

b Activation entropy (J/(mol K)).

c Activation energy (kJ/mol).

d Turnover frequency (mol_H_2__/(mol_cat_·min)).

e Temperature (K).

f Substrate concentration (mM)­s.

To demonstrate the stability of the catalyst (Pd_0.5_Ru_0.5_/AC@1% Co) in the hydrolysis reaction of AB, its reusability was tested by measuring the H_2_ production rate during the consecutive runs of the experiment. For this purpose, the first cycling experiment was initiated at 298 K in the presence of 30 mg of Pd_0.5_Ru_0.5_/AC@1% Co and 100 mM AB. After the release of stoichiometric H_2_ gas (3.0 mol H_2_/mol AB) in the first catalytic cycle, the catalyst was isolated from the reaction solution by centrifugation and dried. The isolated and dried Pd_0.5_Ru_0.5_/AC@1% Co catalyst was reused in AB hydrolysis under the same conditions. This process was repeated for 6 experiments, and the results were evaluated by the initial catalytic activity of Pd_0.5_Ru_0.5_/AC@1% Co in the sequential hydrolysis of AB. At the end of 6 repeated catalytic cycles, it was understood that the catalyst largely maintained its initial efficiency in the AB hydrolysis reaction and provided a high conversion; therefore, it was quite stable and could be reused (Figure 11a). This result indicates that the crystal structure of the catalyst is mostly unchanged, and the PdRuCo NPs maintain their distribution on the support surface throughout the cycles. TOF values for all six consecutive catalytic runs were calculated based on the initial linear regions of the hydrogen evolution profiles shown in Figure 11b. The catalytic activity began to decline gradually after repeated use. The initial TOF in the first cycle was calculated to be 312.8 (mol_H_2__)/(mol_cat_·min) at 298 K. A progressive decrease in TOF values was observed over the subsequent cycles, which could be attributed to partial catalyst deactivation or surface poisoning effects. This decrease is likely due to the increase in metaborate concentration or the decrease in material transfer due to closure of the catalyst surface.

**Figure 11:**
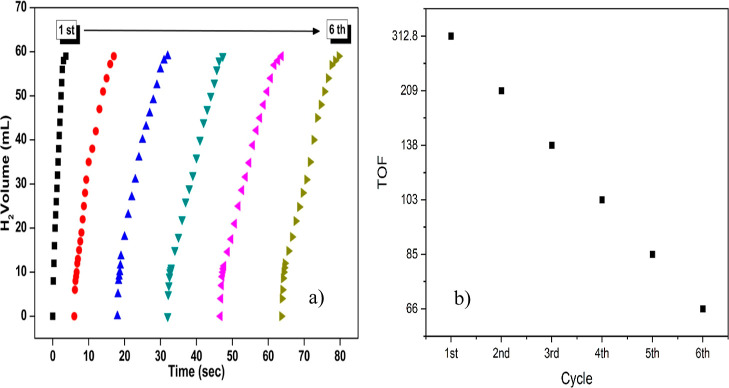
Plots of hydrogen volume versus time showing the durability of the Pd_0_._5_Ru_0_._5_/AC@1% Co catalyst during repeated catalytic lifetime tests: (a) hydrogen generation over 80 s and (b) catalytic performance across multiple cycles.

## 4 Conclusion

In this study, PdRuCo NP supported on AC was successfully synthesized via the impregnation–reduction method for the catalytic hydrolysis of AB. The catalyst was characterized using advanced techniques including SEM, XRD, and XPS. The catalyst composition and reaction conditions were optimized to enhance hydrogen generation performance. Among the tested formulations, the Pd_0_._5_Ru_0_._5_/AC@1% Co catalyst exhibited the highest catalytic activity, with a TOF of 312.8 (mol_H_2__)/(mol_cat_·min) at 298 K. The activation parameters for the hydrolysis of AB were calculated as follows: *E*_a_ = 19.65 kJ/mol, Δ*H* ^#^ = 17.12 kJ/mol, and Δ*S* ^#^ = 184.85 J/ (mol K). These results demonstrate that the Pd_0_._5_Ru_0_._5_/AC@1% Co catalyst, with its simple preparation route, high activity, and good reusability, can serve as a promising candidate for efficient hydrogen production from AB and may contribute significantly to the design of next-generation catalytic systems.
